# Anti-fatigue mechanism of *Dendrobium officinale* pseudobulbs from different growth durations against chronic fatigue syndrome in mice and analysis of their active chemical components

**DOI:** 10.3389/fcell.2026.1797074

**Published:** 2026-04-22

**Authors:** Minda Zhang, Haishen Li, Junhao Dai, Xiaotong Wang, Jian Sun, Jing Xu, Kang Ye, Chuanbao Wang, Long Cai, Zhian Wang, Qingsong Shao, Zhenhao Li

**Affiliations:** 1 Zhejiang Research Institute of Traditional Chinese Medicine Co., Ltd., Hangzhou, China; 2 Zhejiang Provincial Key Laboratory of Resources Protection and Innovation of Traditional Chinese Medicine, Zhejiang Agriculture and Forest University, Hangzhou, China; 3 Zhejiang Engineering Research Center of Rare Medicinal Plants, Jinhua, China; 4 Zhejiang Shouxiangu Botanical Drug Institute Co., Ltd., Hangzhou, China; 5 State Key Laboratory for Development and Utilization of Forest Food Resources, Zhejiang Agriculture and Forest University, Hangzhou, China

**Keywords:** anti-fatigue, bioactive constituents, chronic fatigue syndrome, *Dendrobium officinale*, multi-omics analysis, phytochemical profiling, plasma metabolomics

## Abstract

To investigate the differential efficacy of *Dendrobium officinale* pseudobulb alcohol extracts from varying growth durations against chronic fatigue syndrome (CFS) in mice, elucidate the underlying anti-CFS mechanisms, and identify the pharmacologically active components responsible. An ICR mouse model of CFS was established using the forced swimming test (FST). Behavioral characteristics, physiological phenotypes, biochemical parameters, and plasma metabolomics profiles were assessed. Concurrently, untargeted metabolomics and high-performance liquid chromatography (HPLC) were employed to analyze the chemical constituents of *D. officinale* pseudobulbs from different cultivation periods. Correlation analysis between the identified anti-CFS characteristic metabolites in plasma and the pseudobulb components was performed to predict the potential active ingredients against CFS in *D. officinale* pseudobulbs. Compared with the one-year-cultivated *D. officinale* extract (1DOE), the three-year-cultivated *D. officinale* extract (3DOE) significantly reduced anal temperature, prolonged forced swimming time, decreased liver index and plasma lactate dehydrogenase (LDH) levels, lowered cAMP levels and the cAMP/cGMP ratio, and mitigated renal inflammatory infiltration in model mice. Plasma metabolomic pathway analysis indicated that 3DOE extract alleviated energy metabolism dysregulation by enhancing energy metabolism and promoting oxidative utilization of fatty acids and proteins. Characteristic metabolites included arachidonic acid, L-5-oxoproline, pyroglutamic acid, D-(+)-malic acid, and PC (20:5/22:5). Correlation analysis between key metabolites and pseudobulb bioactive constituents revealed that flavonoids, polyphenols, lignans, terpenoids, and bibenzyls, which are significantly enriched in 3DOE, constitute the pharmacodynamic basis for the anti-CFS efficacy. These findings provide a scientific foundation for applying perennial *D. officinale* in the management of fatigue-related disorders, including CFS.

## 1 Introduction

*Dendrobium officinale* Kimura & Migo (Orchidaceae) is a naturally rare and precious traditional Chinese medicine widely used as a tonic for enhancing immunity and benefiting stomach qi. Modern research indicates that *D. officinale* contains various active components, such as Dendrobium polysaccharides, alkaloids, polyphenols, flavonoids, and bibenzyls. These constituents exhibit pharmacological effects including anti-inflammatory, antioxidant, anticancer, immunoenhancing, anti-fatigue, and neuroprotective activities (Xu et al., 2022). Polysaccharides represent the primary pharmacologically active constituents of *D. officinale* and are frequently employed in studies investigating its therapeutic effects. They are primarily extracted from the pseudobulb of *D. officinale*, with a yield of about 30%. Macromolecular polysaccharides derived from *D. officinale* exert anti-fatigue effects by enhancing the community structure of the intestinal microbiota, accelerating fatigue-related metabolism *in vivo*, and improving immunoregulatory activity (Cai et al., 2023; Wei et al., 2017).

Myalgic Encephalomyelitis/Chronic Fatigue Syndrome (ME/CFS) is a complex multisystem disease characterized by post-exertional malaise (PEM), pervasive fatigue, cognitive impairment, sleep disturbances, orthostatic intolerance, myalgia, and neuroendocrine-immune dysfunction. Affecting approximately 10% of the population, this condition severely compromises quality of life and work capacity. To date, neither definitive diagnostic biomarkers nor established therapeutic frameworks exist. Substantial evidence implicates post-infectious dysregulation across neurological, immune, endocrine, and metabolic systems, coupled with oxidative stress; however, the precise etiology and pathophysiology remain elusive (Shankar et al., 2024). This knowledge gap stems largely from disease heterogeneity, where divergent molecular damage patterns drive varied clinical presentations. Research confirms that ME/CFS involves multisystem abnormalities, including impaired energy metabolism, mitochondrial dysfunction, neuroendocrine perturbations, and gastrointestinal microbiota alterations (Stallmach et al., 2024).

Traditional Chinese Medicine (TCM), as a medical system with a long history and widespread application, exhibits unique advantages in treating CFS due to its integrated therapeutic effects characterized by “multi-component, multi-target, and multi-system” actions. Key anti-fatigue bioactive components primarily include polysaccharides, polyphenols, flavonoids, and terpenoids. Polyphenols and flavonoids, characterized by multiple hydroxyl groups, possess high antioxidant activity, which likely constitutes the primary mechanism underlying their anti-fatigue capacity (Zhang et al., 2024; Zhang et al., 2021). Terpenoids isolated from Chinese medicinal herbs are also representative anti-fatigue constituents, and the reduction of metabolite accumulation may represent their underlying mechanism (Yu et al., 2023).

To systematically decipher the structure-activity relationship between the complex chemical composition of *D. officinale* and its anti-fatigue effects, this study established a CFS mouse model via an overloaded forced swimming paradigm. This model was subsequently applied to evaluate the differential anti-CFS efficacy of *D. officinale* samples across distinct growth years. Specifically, 1-year material was utilized as a juvenile baseline, whereas 3-year material was selected to represent a relatively mature perennial stage. This comparison aimed to ascertain whether the transition from an early to a mature cultivation phase is accompanied by enhanced anti-fatigue efficacy and a broader accumulation of secondary metabolites. By doing so, this study sought to explore mechanism-related metabolic pathways affected by treatment and characterize the bioactive constituent profiles conferring CFS resistance in multi-year cultivated *D. officinale*.

## 2 Materials and methods

### 2.1 Plant material collection and extract preparation

*Dendrobium officinale* cultivated on trees in Pan’an County, Zhejiang Province, China, were uniformly harvested in December, which complies with the collection period and general post-harvest processing guidelines specified in the Chinese Pharmacopoeia. Based on growth duration, the plants were divided into two groups: 1DOE (juvenile) and 3DOE (mature perennial). The harvested stems were washed, sliced, dried at 60 °C, ground into powder, passed through a No. 3 sieve, and stored at 4 °C until use.

The extract for animal studies was prepared by reflux extraction using absolute ethanol (≥99.7%, anhydrous) at 100 °C with a solid-to-solvent ratio of 1:20 (w/v) for 5 h. The resultant extract was concentrated under reduced pressure to a final volume of 200 mL, yielding a *D. officinale* ethanol extract solution with a mass concentration of 0.1 g/mL.

### 2.2 Animals

A total of 100 six-week-old male ICR mice (Specific Pathogen Free [SPF] grade) weighing 18–21 g were supplied by Hangzhou Medical College (Animal Use License No. SYXK (Zhe)2018-0011). Mice were housed in a barrier-sustained animal facility maintained at 20 °C–26 °C with 40%–70% relative humidity, a 12-h light/dark cycle, and ventilation ≥22 air changes per hour. Prior to experimentation, all mice underwent a 1-week acclimation period with *ad libitum* access to standard pelleted diet and water. The experimental protocol was approved by the Laboratory Animal Care Committee of Zhejiang Institute of TCM Co., Ltd. (Approval No. 20220501) and complied with the ethical requirements of the Zhejiang Laboratory Animal Management Regulations.

### 2.3 Establishment of the CFS model

A CFS mouse model was established through sustained overload swimming based on previously reported protocols (Liu et al., 2011; Yin et al., 2021). This paradigm reproduces key behavioral and physiological features of chronic fatigue, including reduced endurance, weight loss, and abnormal serum markers. Mice in the normal control group (Normal) did not undergo swimming, whereas the model group underwent forced swimming in a 30 °C ± 1 °C water maze once daily, 5 days per week, for 6 weeks. Duration was incrementally extended from 20 min/day (Week 1) to 120 min/day (Week 6). Post-swimming, mice were thoroughly dried using a warm-air dryer to prevent hypothermia.

### 2.4 Experimental design

A total of 100 mice were randomly assigned to four experimental groups, namely, normal, model, 1DOE, and 3DOE, with 25 mice in each group. The treatment groups received daily oral administration of the test substance at 2 g/kg body weight (20 mL/kg dosing volume) for seven consecutive days per week over 6 weeks. Body weights were recorded every Monday and Friday. On the day following the final swimming session: fresh fecal samples were collected and snap-frozen; anal temperature was measured; physical endurance was assessed via weighted swimming test with a 2-g tail load; and blood glucose levels were determined using a portable glucose meter. The next day, blood was collected via retro-orbital puncture with EDTA-2K anticoagulant for blood cell analysis. Residual whole blood was centrifuged at 3,500 rpm for 15 min to isolate plasma. Plasma concentrations of cAMP (cyclic adenosine monophosphate), cGMP (cyclic guanosine monophosphate), ALT (alanine aminotransferase), AST (aspartate aminotransferase), LDH (lactate dehydrogenase), and BUN (blood urea nitrogen) were quantified. Residual plasma and fecal samples were separately submitted for metabolomic analysis. Mice were euthanized by cervical dislocation under anesthesia, followed by collection and weighing of thymus, spleen, liver, kidneys, and colon. Tissues were fixed in 10% neutral buffered formalin for histopathological examination with H&E staining.

### 2.5 Exhaustive swimming

The exhaustive swimming test was performed with minor modifications according to previously reported methods (Liu et al., 2011; Yin et al., 2021). A 2-g weight was affixed to the base of the mouse tail using medical-grade twine. Mice were gently placed in individual constant-temperature water tanks (30 °C ± 1 °C), and total swimming duration until exhaustion was recorded. Exhaustion was defined as the inability to resurface within 3 s after submersion. Mice were promptly retrieved upon meeting this criterion. Swimming durations exceeding 10 min (600 s) were recorded as 600 s.

### 2.6 Biochemical measurements

Plasma concentrations of ALT, AST, LDH, BUN, cAMP, and cGMP were quantified using commercial kits following manufacturers’ instructions. The cAMP and cGMP ELISA kits were from Land-Biotech Co., Ltd. (Shanghai, China; Lot No. G20221028MR and G20221106MN, respectively). ALT, AST, LDH, and BUN assay kits were purchased from Jiancheng Bioengineering Institute (Nanjing, China; Lot No. 20221124, 20221123, 20221122, and 20221124, respectively). Hematoxylin and Eosin (H&E) staining reagents were sourced from Fine-Biological Technology Co., Ltd. (Lot No. 20201116).

### 2.7 Metabolomic profiling of *Dendrobium officinale* and plasma samples

Collected samples were thawed on ice, and metabolites were extracted with 50% methanol solution. Briefly, 100 mg of sample was extracted with 1 mL of precooled 50% methanol, vortexed for 1 min, and incubated at room temperature for 10 min; the extraction mixture was then stored overnight at −20 °C. After centrifugation at 4,000 g for 20 min, the supernatants were transferred into new 96-well plates and stored at −80 °C before LC-MS analysis.

All samples were acquired by the LC-MS system according to the sample queue. Chromatographic separations were performed on a Vanquish Flex UHPLC system (Thermo Fisher Scientific, Bremen, Germany). An ACQUITY UPLC T3 column (100 mm × 2.1 mm, 1.8 µm; Waters, Milford, United States) was used for reversed-phase separation. The column temperature was maintained at 35 °C with a flow rate of 0.4 mL/min. The mobile phase consisted of solvent A (water with 0.1% formic acid) and solvent B (acetonitrile with 0.1% formic acid). The gradient elution program was: 0–0.5 min, 5% B; 0.5–7 min, 5%–100% B; 7–8 min, 100% B; 8–8.1 min, 100%–5% B; 8.1–10 min, 5% B (followed by 2-min column equilibration).

Metabolites eluted from the column were detected using a Q-Exactive high-resolution tandem mass spectrometer (Thermo Scientific). The instrument alternated between positive and negative ion modes. Full MS scans (m/z 70–1050) were acquired at 70,000 resolution with an AGC target of 3e6 and maximum injection time of 100 ms. MS/MS spectra were collected in data-dependent acquisition (DDA) mode (top three ions) at 17,500 resolution with an AGC target of 1e5 and maximum injection time of 80 ms. Quality control samples were interspersed throughout the run to evaluate system stability.

### 2.8 Determination of chemical components in *Dendrobium officinale*

Simultaneous quantification of six flavonoids in *dendrobium officinale* using HPLC-QAMS with dual-wavelength detection: vicenin-1, vicenin-2, schaftoside, isoschaftoside, rutin, and naringenin (Ding et al., 2021). Chromatographic separation was performed on an Agilent Eclipse XDB-C18 column (250 mm × 4.6 mm, 5 μm) with a mobile phase consisting of acetonitrile (A) and 0.2% aqueous formic acid (B). The gradient elution program was as follows: 0–10 min, 10%–15% A; 10–35 min, 15%–18% A; 35–45 min, 18%–30% A; 45–60 min, 30%–45% A. The flow rate was 1.0 mL/min, the column temperature was maintained at 30 °C, and the injection volume was 10 μL. Detection wavelengths were set at 340 nm for vicenin-1, vicenin-2, schaftoside, isoschaftoside, and rutin, and at 290 nm for naringenin.

### 2.9 Statistical analysis

For conventional quantitative indices (physiological, biochemical, and histological), inter-group differences were evaluated by Student's *t*-tests in GraphPad Prism 9.0, with statistical significance defined as *P* < 0.05. Metabolomics-related preprocessing and multivariate analyses (PCA and supervised PLS-DA) were conducted exclusively in R with the metaX workflow. In the untargeted plasma metabolomics analysis, univariate *P* values from Student's *t*-tests were corrected using the Benjamini–Hochberg procedure to generate adjusted *q* values. Differential metabolites were identified based on adjusted q values from univariate analysis and VIP scores from the PLS-DA model. Unless otherwise stated, key findings for plasma metabolites satisfied both VIP > 1 and *q* < 0.05. Detailed raw *P*, adjusted *q*, and VIP values are provided in the Supplementary Material.

## 3 Results

### 3.1 General state observation

**FIGURE 1:**
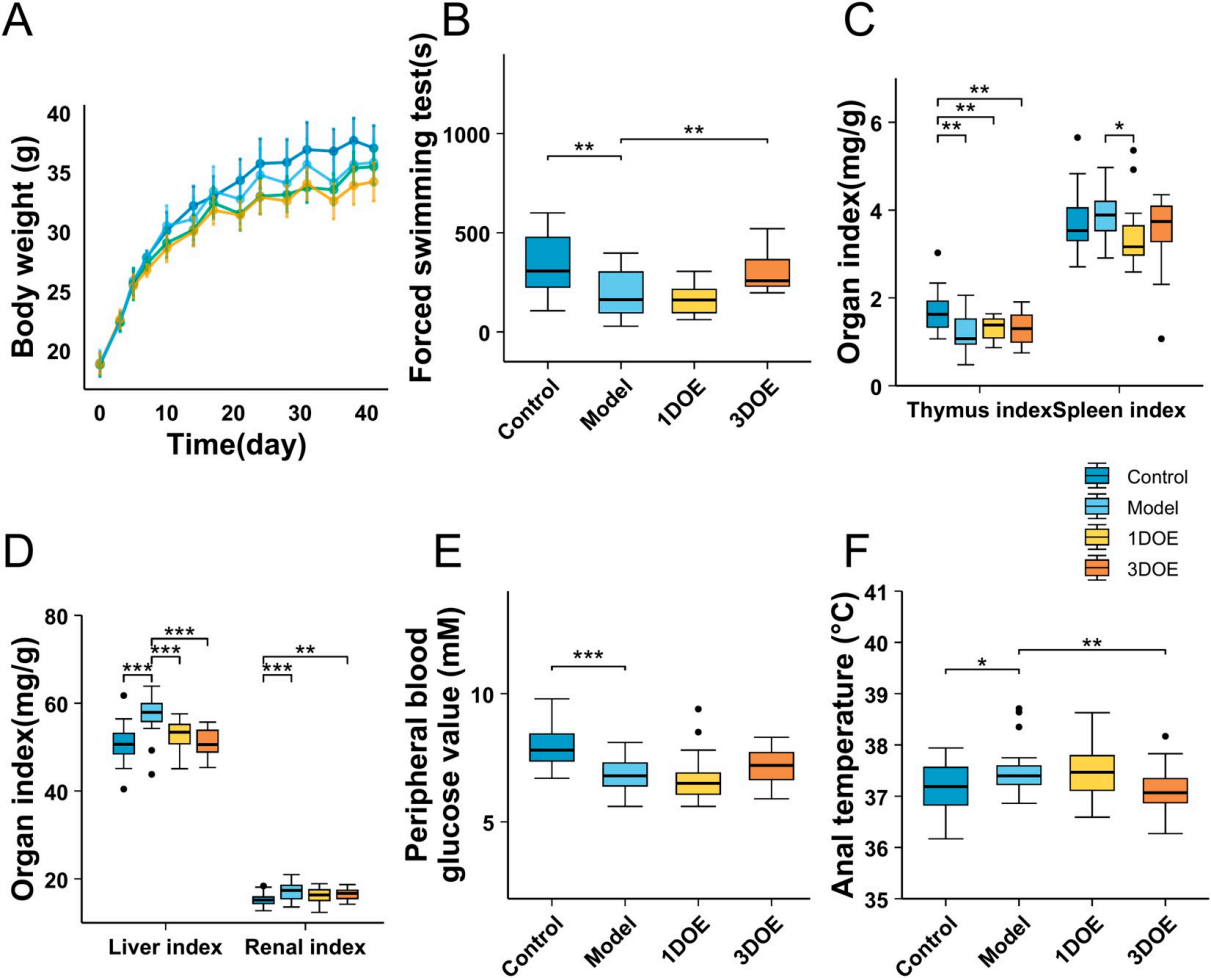
Physiological and behavioral parameters in CFS mice treated with 1DOE and 3DOE. **(A)** Body weight change. **(B)** Forced swimming time. **(C, D)** Organ indices. **(E)** Blood glucose level. **(F)** Anal temperature. *P* < 0.05, **P < 0.01, ***P < 0.001 vs. Model.

During the modeling period, the body weight gain rate was significantly lower in the model group and both treatment groups compared to the normal group. Compared with the normal group, the model group showed significant decreases in forced swimming time, thymus index, and peripheral blood glucose levels, along with significant increases in liver index and anal temperature. No significant changes were observed in spleen index or renal index compared to the normal group. After treatment, both the 1DOE and 3DOE extracts significantly decreased the liver index in the model group mice. The 1DOE extract did not significantly affect forced swimming time. In contrast, the 3DOE extract significantly decreased anal temperature and increased forced swimming time in the model group (Figure 1). Because anal temperature was elevated in the model group relative to the normal group and was reduced after 3DOE treatment, this change may suggest partial normalization of thermoregulatory disturbance under chronic fatigue stress rather than a nonspecific suppression of basal metabolism. Recent evidence indicates that core body temperature is closely related to exercise capacity (Xi et al., 2025). From this perspective, the reduction in anal temperature after 3DOE administration may reflect alleviation of thermoregulatory burden and is consistent with the broader improvement observed in plasma metabolomic profiles.

### 3.2 Detection results of plasma ALT, AST, LDH, BUN, cAMP, cGMP, and histopathological examination

**FIGURE 2:**
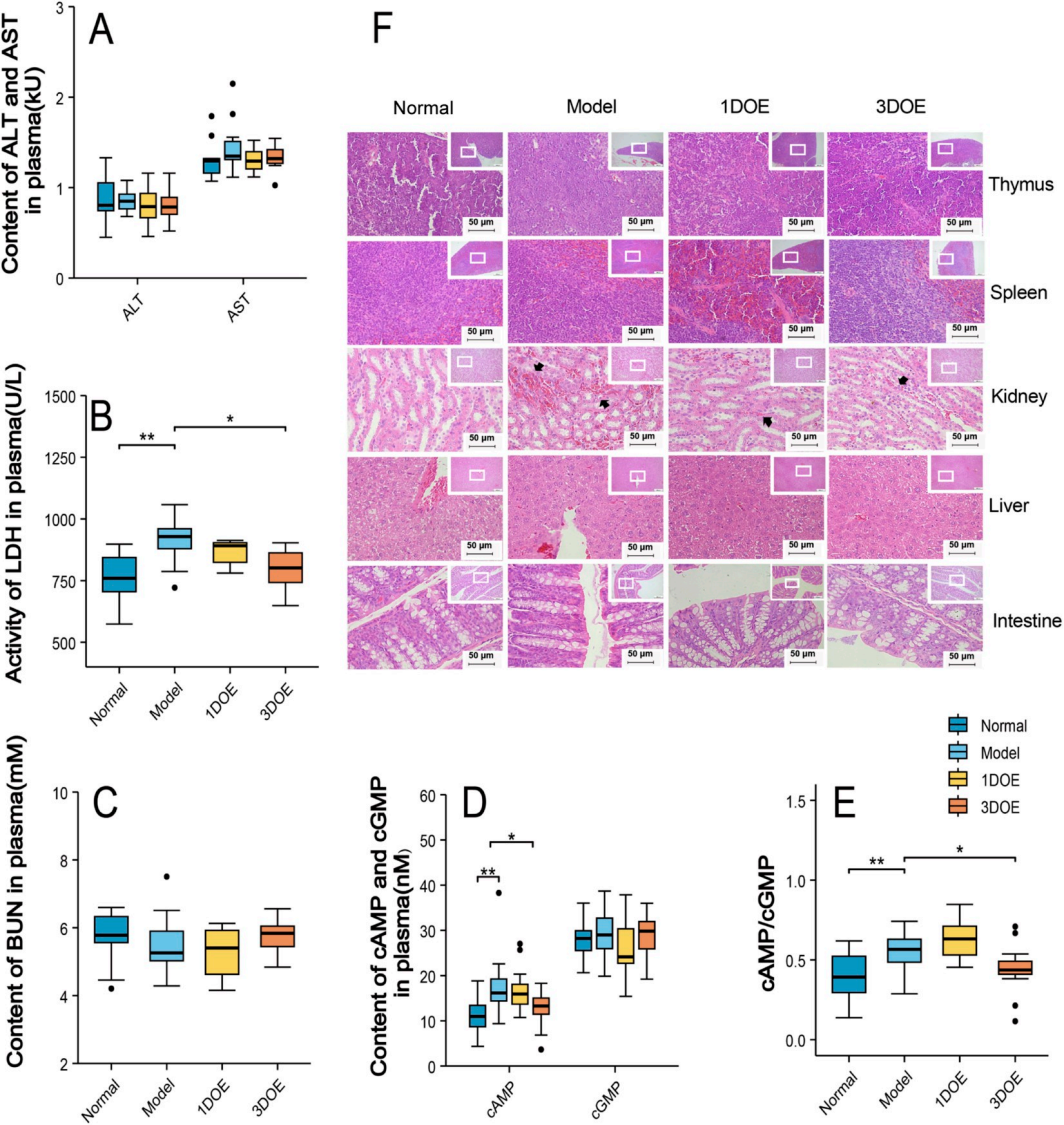
Plasma biochemical parameters and histopathological changes. **(A–C)** Plasma ALT, AST, LDH, and BUN levels. **(D, E)** Plasma cAMP and cGMP levels. **(F)** Representative H&E-stained histopathological images. *P* < 0.05, **P < 0.01, ***P < 0.001 vs. Model.

Compared with the normal group, the model group showed significantly elevated plasma levels of LDH, cAMP, and the cAMP/cGMP ratio, as well as mild inflammatory cell infiltration and hemorrhage in renal tissue. There were no significant differences in plasma ALT, AST, or BUN levels between the model group and the normal group. Administration of the 3DOE extract significantly reduced plasma LDH, cAMP, and the cAMP/cGMP ratio in the model group mice and attenuated renal inflammatory infiltration (Figure 2).

### 3.3 Plasma metabolomics results

**FIGURE 3:**
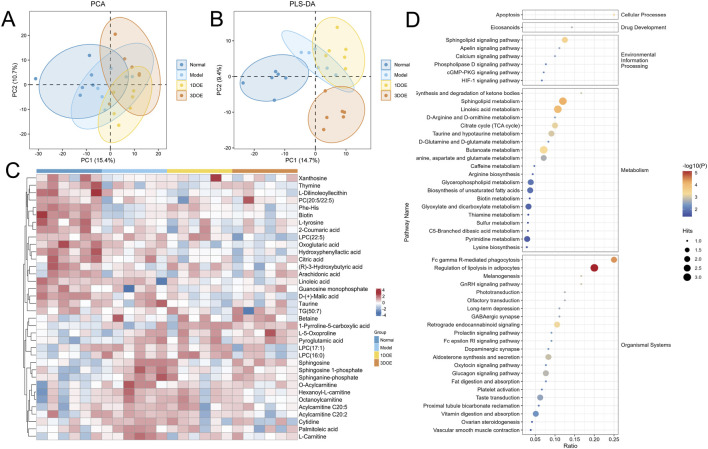
Plasma metabolomics analysis of CFS mice. **(A, B)** PCA and PLS-DA score plots. **(C)** Heatmap of differential metabolites. **(D)** KEGG pathway enrichment plot. Metabolites, in which rows represent metabolites, columns represent samples, and the color scale indicates relative abundance. **(D)** KEGG pathway enrichment bubble plot of the metabolites reversed by *Dendrobium officinale* treatment, where bubble size represents the number of matched metabolites and bubble color reflects enrichment significance.

Partial least squares-discriminant analysis (PLS-DA) was performed to model and maximize the separation between groups for identifying differential metabolites (Figure 3). The PLS-DA score plot showed that samples did not separate well in the plane defined by the first two principal components (PC1 vs PC2), but achieved clear separation when the third principal component (PC3) was included. The model parameters were R2X = 0.527, R2Y = 0.987, and Q2 = 0.747. 36 differential metabolites were identified (Supplementary Table S1). The differential metabolites primarily comprised carnitines, lysophosphatidylcholines (LPCs), sphingolipids, organic acids, amino acids and derivatives, fatty acids, and nucleosides. Compared with the normal group, the model group had 18 upregulated and 18 downregulated metabolites. Compared to the control group, the model group showed: Increased levels of acylcarnitines (e.g., hexanoyl-L-carnitine, octanoyl-L-carnitine, L-carnitine) and sphingolipid metabolites (e.g., sphingosine, sphinganine 1-phosphate). Decreased levels of organic acids (e.g., citric acid, malic acid, α-ketoglutaric acid) and specific amino acid/nucleic acid metabolites (e.g., thymine, xanthosine). After treatment, the dysregulated levels of all specified metabolites—including acylcarnitines (hexanoyl-L-carnitine, octanoyl-L-carnitine), sphingosine-related compounds, LPC(16:0), linoleic acid, arachidonic acid, and phosphatidylcholine PC(20:5/22:-5) — were reversed.

Twenty-two of these dysregulated metabolites showed reversed trends in the *D. officinale* treatment group, demonstrating that *D. officinale* ameliorated metabolic disorders in mice with exercise-induced fatigue.

Enrichment analysis of the differential metabolites using the KEGG database revealed that the effects of *D. officinale* were primarily enriched in apoptosis-related processes. This suggests modulation of multiple signaling pathways, including the Sphingolipid signaling pathway, Calcium signaling pathway, HIF-1 signaling pathway, and cGMP-PKG signaling pathway. These pathways are known to regulate critical biological processes, including cell proliferation, apoptosis, inflammatory responses, angiogenesis, and neural signaling transduction. Additionally, enrichment analysis identified significant perturbation in core metabolic pathways, including Ketone body metabolism, Citrate cycle (TCA cycle), Alanine, aspartate and glutamate metabolism, Pyrimidine metabolism, and Glycerophospholipid metabolism.

### 3.4 Non-targeted metabolomics of *Dendrobium officinale* at different cultivation period

**FIGURE 4:**
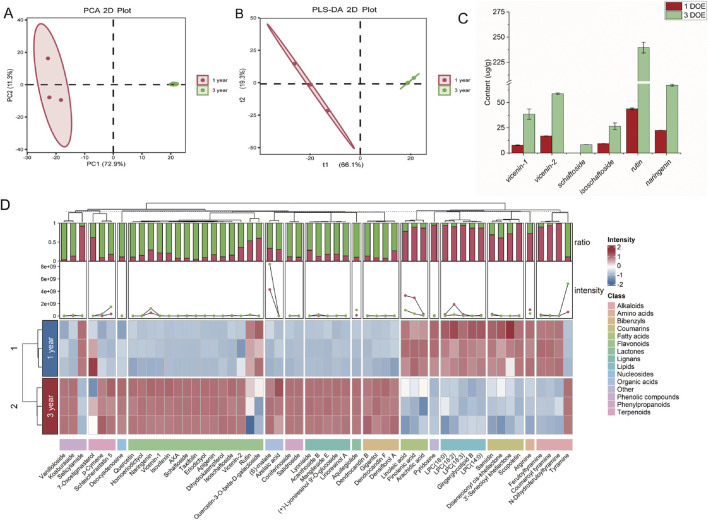
Comparative metabolite profiling of 1-year and 3-year *Dendrobium officinale*. **(A, B)** PCA and PLS-DA score plots. **(C)** HPLC quantification of representative flavonoids. **(D)** Heatmap showing the distribution of differential constituents across chemical classes.

Using positive and negative ion modes in tandem mass spectrometry (MS/MS), 1,647 and 1,383 metabolic features were detected, respectively. Metabolite diversity was higher in 3DOE compared to 1DOE. Principal component analysis (PCA) showed clear separation between the 1DOE and 3DOE cultivation groups, demonstrating substantial differences in overall chemical composition (Figures 4A,B). To explore these compositional differences, PLS-DA was further used to screen candidate discriminating features with VIP > 1. These features were then putatively annotated by integrating accurate mass, MS/MS fragmentation patterns, retention behavior, and database/literature matching. Supplementary Table S2 summarizes 53 putatively annotated candidate constituents, detailing the overall chemical composition and class-wide shifts associated with cultivation age.

The 53 differential metabolites were classified into thirteen major classes: flavonoids, alkaloids, coumarins, phenylpropanoids, bibenzyls, terpenoids, lipids, fatty acids, organic acids, phenolic, lignans, and O-glycosyl compounds. Flavonoids represented the most abundant class, followed by alkaloids and lipids.

Heatmap analysis revealed significantly enriched accumulation of flavonoids (such as Vicenin-1, Vicenin-2, AXA, Isovitexin, Schaftoside), terpenoids, O-glycosyl compounds, phenylpropanoids, bibenzyls, phenolic, lignans, and organic acids in 3DOE, Conversely, alkaloids, coumarins, lipids, and fatty acids showed predominant accumulation in 1DOE (Figure 4D). To validate these findings, the established chromatographic method was employed to quantify six flavonoids—vicenin-1, vicenin-2, schaftoside, isoschaftoside, rutin, and naringenin—with HPLC results further demonstrating significant enrichment of rutin-representative flavonoids in 3DOE pseudobulbs (Figure 4C).

### 3.5 Multi-omics correlation analysis

**FIGURE 5:**
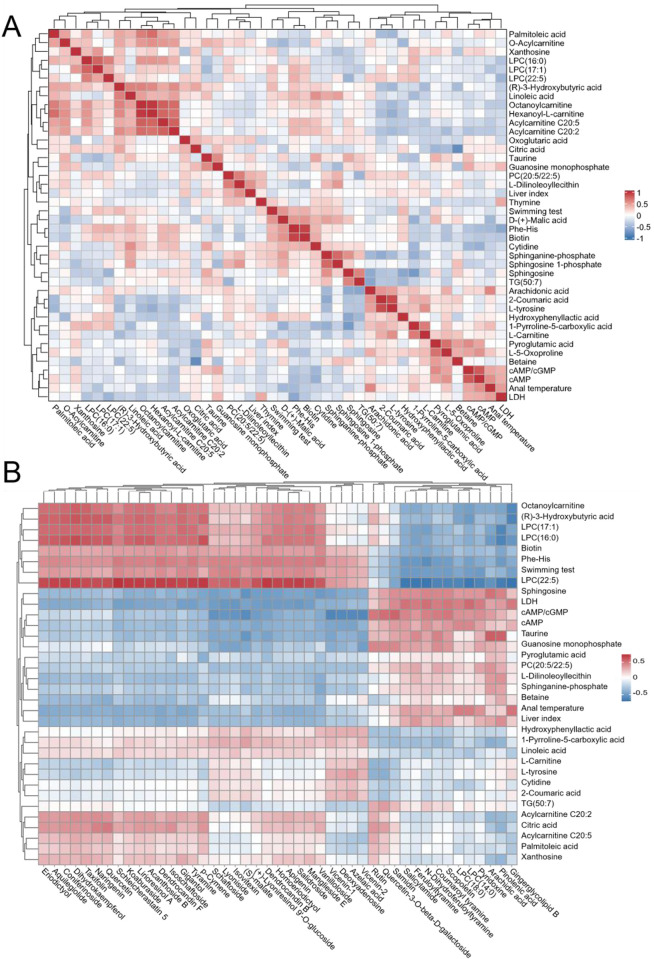
Analysis of Characteristic Components in *Dendrobium officinale* for Treating CFS. **(A)** Correlation analysis among physiological indicators; **(B)** Correlation analysis between physiological indicators and characteristic components in 3DOE.

Based on the aforementioned data, we further conducted Spearman correlation analysis to determine the correlations between plant components related to therapeutic effects and murine general status indicators, biochemical indices, and metabolites (Figure 5). In our experiment, general status indicators and biochemical indices showing significant differences among the normal group, model group, and treatment groups included cAMP/cGMP, cAMP, Anal temperature, LDH, Swimming test, Liver index. According to the correlation analysis results, plasma metabolites significantly correlated with these included Arachidonic acid, L-5-Oxoproline, Pyroglutamic acid, D-(+)-Malic acid, plasma protein C (PC) (20:5/22:5), Phe-His, lysophosphatidylcholine (LPC) (22:5), Octanoylcarnitine, (R)-3-Hydroxybutyric acid, LPC (16:0), LPC (17:1), L-tyrosine, Sphingosine, Taurine. And LPC (22:5), Octanoylcarnitine, (R)-3-Hydroxybutyric acid, LPC (16:0), LPC (17:1) showing the most significant correlations with plant components.

According to the correlation results, the phytochemical components most strongly associated with the anti-fatigue efficacy of *D. officinale* primarily include flavonoids (such as eriodictyol, naringenin, and quercetin), polyphenols (koaburaside, gigantol, and vanilloloside), the lignan (+)-lyoniresinol 9′-O-glucoside, the neolignan acanthoside B, the terpenoid p-cymene, the sesquiterpene schleicherastatin 5, the bibenzyl dendrocandin B, and phenylpropanoids.

## 4 Discussion

### 4.1 Analysis of the mechanism underlying the therapeutic effects of *Dendrobium officinale* on CFS

In our study, supplementation with *D. officinale* extract synergistically modulated multiple energy metabolism pathways to a certain extent, thereby stabilizing energy supply in a CFS mouse model. In the LC-MS analysis of plasma metabolites from the CFS mouse model group, 36 differential metabolites were identified, including dysregulation of acylcholines, sphingolipids, carnitines, dipeptides, triglycerides, α-ketoglutarate, purines, and other components. Notably, most categories of these differential metabolites have been reported in previous metabolomic studies of ME/CFS (Che et al., 2022; Germain et al., 2020; Nagy-Szakal et al., 2018; Naviaux et al., 2016; Stallmach et al., 2024), which supports the validity of the animal model. The metabolic pathways associated with these metabolites include synthesis and degradation of ketone bodies, the tricarboxylic acid (TCA) cycle, alanine metabolism, aspartate and glutamate metabolism, pyrimidine metabolism, and glycerophospholipid metabolism. In the mouse model, exposure to stressful environments induced TCA cycle dysfunction, which may partially explain fatigue symptoms in ME/CFS. Under glucose-depleted conditions, organisms activate compensatory energy pathways including glycolysis, fatty acid β-oxidation, and proteolysis (Zhang et al., 2017). In this study, the model group exhibited significantly reduced levels of α-ketoglutarate, citrate, and malate compared to the Normal group. α-Ketoglutarate is associated with the TCA cycle, indicating their deficiency in adenosine triphosphate (ATP) production, which serves as a holistic reflection of bodily energy metabolism (Hannun and Obeid, 2018). In the treatment group, plasma concentrations of fatty acid metabolism-related components such as carnitine, linoleic acid, and triglycerides decreased in plasma, while increases in pyroglutamic acid and 1-pyrroline-5-carboxylic acid signify that *D. officinale* achieves energy balance by regulating lipid and protein homeostasis. Existing studies have revealed that plasma metabolomic enrichment results from ME/CFS patients demonstrate imbalances in energy metabolism, amino acid metabolism, nucleotide metabolism, redox metabolism, lipid metabolism, and neurotransmitter metabolism (Prins et al., 2006). In the kidneys, arginine is utilized for synthesizing multiple metabolites including polyamines and creatine, while enzymes involved in the urea cycle are expressed in renal tissue. Glutamine plays a central role in renal metabolism by facilitating nitrogen elimination, acid-base balance regulation, and gluconeogenesis (Knol et al., 2024). Concurrently, proline interconverts with glutamate and arginine via the pivotal intermediate P5C/glutamate-γ-semialdehyde (GSA), thereby connecting the TCA cycle, urea cycle, and proline metabolic pathways. This network further participates in regulating cell growth, redox homeostasis, ATP generation, and immune modulation (Jones, 1985; Kuo et al., 2020). Existing clinical studies indicate that plasma phenylalanine (Phe) and histidine (His) levels exhibit strong positive correlations with hyperlipidemia in healthy cohorts (Chen et al., 2020). In our treatment group, restorative elevations of Phe and His occurred concomitantly with increased plasma lipid concentrations. These coordinated changes facilitated stabilization of multiple energy metabolism pathways.

Excessive fatigue may induce inflammation *in vivo*. The results of this study demonstrate that supplementation with *D. officinale* extract reduces inflammatory levels and alleviates pain. Multiple factors including insulin and growth factors have been proven to activate sphingosine kinases (SKs) through phosphorylation mechanisms, leading to intracellular formation of sphingosine-1-phosphate (S1P) from sphingosine (Sph). S1P must translocate extracellularly to interact with S1P receptors (S1PRs). S1P can also be released from the plasma membrane into the bloodstream, circulating at relatively high levels. S1PRs transmit signals to key downstream targets such as serine/threonine protein kinase AKT, RHO, and the Ras-ERK and tyrosine protein kinase JAK-signal transducer and activator of transcription (STAT) pathways (Hannun and Obeid, 2018), S1P signaling is involved in nearly all major aspects of cell biology, including roles in cell growth, death, inflammation, immune responses, cell adhesion and migration, angiogenesis, nutrient uptake, metabolism, and nociceptive pain (Dong et al., 2024; Hannun and Obeid, 2008; Spiegel and Milstien, 2011), Studies indicate that S1P plays a critical role in inflammatory responses by promoting the production of inflammatory cytokines, particularly interleukin-6 (IL-6) and CC-chemokine ligand 5 (CCL5), in response to tumor necrosis factor (TNF) and IL-1, as well as through the cPLA2–cyclooxygenase 2 (COX-2) pathway. *D. officinale* extract may exert anti-CFS effects via the S1P/S1PR signaling pathway. Concurrently, guanosine monophosphate (GMP) was significantly reduced in the treatment group. The NO/cGMP signaling pathway has been proven to mediate pain transmission and processing (Li et al., 2023). The main process in the NO/cGMP signaling pathway in cells involves NO activating soluble guanylate cyclase (sGC), which leads to subsequent production of cyclic guanosine monophosphate (cGMP). cGMP then activates cGMP-dependent protein kinase (PKG), resulting in the activation of multiple targets such as the opening of ATP-sensitive K^+^ channels (K^+^ATP) (Schmidtko et al., 2008). This process is similarly associated with pain generation, and it is hypothesized that *D. officinale* extract alleviates chronic pain in CFS patients through the NO/cGMP signaling pathway.

**FIGURE 6:**
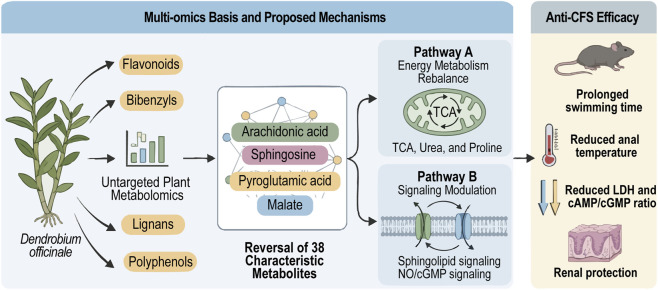
Proposed pharmacological mechanism of *Dendrobium officinale* against CFS.

Building on the pathway-level enrichment and correlation results, we propose an integrated pathway-level framework (Figure 6) linking plasma metabolomics with plant non-targeted metabolomics and phytochemical profiling. This integrated analysis jointly screened key candidate compounds, suggesting that constituents relatively enriched in 3DOE, especially flavonoids, polyphenols, lignans, terpenoids, and bibenzyls, were associated with reversal of representative plasma metabolites such as arachidonic acid, pyroglutamic acid, D-(+)-malic acid, and PC (20:5/22:5). These associations corresponded to modulation of energy-metabolism-related pathways together with sphingolipid- and NO/cGMP-related signaling changes, ultimately accompanying improved endurance and attenuation of inflammatory/metabolic disturbance in the model mice.

Several limitations should be noted. First, the 1DOE and 3DOE groups were compared under the same total-extract dosing framework based on extract mass rather than on matched doses of individual active constituents; therefore, the observed metabolomic differences are interpreted as overall compositional effects of extracts from different cultivation years under an equivalent extract dose rather than as effects attributable to a single standardized compound dose. Second, the current manuscript provides pathway-level support from metabolomic enrichment and correlation analyses, but does not yet provide direct validation of key proteins in the sphingolipid or NO/cGMP pathways. Future work may further address these issues by normalizing representative active constituents, assessing dose-response relationships of defined markers, and incorporating targeted validation assays and additional phenotypic readouts.

### 4.2 Characterization of bioactive constituents in *Dendrobium officinale* and their therapeutic efficacy against CFS

Modern pharmacological studies indicate that contemporary traditional Chinese medicine acts in treating CFS by regulating immune dysfunction, acting as antioxidants, and ameliorating abnormal activities in energy metabolism disorders (Zhang et al., 2022). The pseudobulbs of *D. officinale* contain abundant polysaccharides (Oskouei et al., 2023), it shows a variety of beneficial effects, such as anti-oxidation and anti-inflammation. Compared to 1DOE, 3DOE showed significant enrichment in flavonoids, phenylpropanoids, bibenzyls, terpenoids, and phenols. Among these compounds, flavonoids accounted for the largest proportion with the highest variety of differential compounds. Correlation analysis with components in plasma anti-CFS-related pathways demonstrated that the most significantly fatigue-alleviating components in *D. officinale* were mainly eriodictyol, naringenin, and quercetin. Flavonoids are widely present in the roots, stems, and leaves of Dendrobium species and exhibit anti-inflammatory and antioxidant effects (Wang, 2021). These bioactive compounds may alleviate CFS symptoms by suppressing mast cell-mediated neuroinflammation (Calis et al., 2020). Research demonstrates that the flavonoid content in *D. officinale* gradually increases with growth years, consistent with our research results (Yuan et al., 2022). Phenylpropanoids are derived from the six-carbon aromatic phenyl group and the three-carbon propene tail, form a large class of important secondary metabolites commonly found in plants, including coumarins, lignans, flavonoids, etc (Vogt, 2010). Rutin, as a representative active flavonoid component, has been widely studied for its anti-inflammatory and antioxidant effects (Azeem et al., 2024). Both HPLC and metabolomic results demonstrated its significant accumulation in 3DOE. Coumarins have rarely been reported in studies of *D. officinale* (Wu et al., 2023). We discovered abundant coumarin compounds in 3DOE through mass spectrometry fragmentation, and for the first time identified four coumarin components enriched in perennial *D. officinale*, including Scopoletin, Samidin, 3'-Senecioyl khellactone, and Disenecionyl cis-khellactone. These flavonoid components have all been proven to possess good anti-inflammatory activity, anti-tumor effects, neuroprotective effects, immune regulation, etc (Alkorashy et al., 2020; Antika et al., 2022; Khan et al., 2014; Park et al., 2023). We found that amide alkaloids are the main differential alkaloids in *D. officinale*. Notably, the content of Tyramine was significantly enriched in the pseudobulbs of 3DOE. Previous reports have demonstrated that Tyramine possesses pharmacological effects including anti-inflammatory activity, antioxidant effects, and inhibition of α/β-glucosidase activity (Duan et al., 2022; Lin et al., 2006; Xie et al., 2014). Bibenzyl components are also one of the primary bioactive constituents abundantly discovered in *D. officinale*, exhibiting pharmacological actions such as antioxidant, anti-tumor, anti-diabetic, and neuroprotective effects (He et al., 2020). These compounds were similarly significantly enriched in the pseudobulbs of 3DOE, with representative constituents such as Dendrocandin B, Dendrocandin F, and Densiflorol A. In summary, these data indicate that the enhanced therapeutic efficacy of the 3-year material relative to the 1-year material is associated with a broader accumulation of secondary metabolites, particularly flavonoids and related phenolics. This compositional advantage may contribute to the observed symptomatic improvements in the CFS model. Since the current evaluation is limited to these two specific growth stages, further investigations incorporating broader temporal gradients are needed to fully elucidate the dynamic accumulation patterns of these bioactives.

## 5 Conclusion

The extract from 3DOE pseudobulbs demonstrated significant anti-CFS effects in mice, manifested as enhanced locomotor activity and reduced inflammation. The anti-CFS effects of the *D. officinale* extract are primarily mediated by modulating specific energy metabolism pathways. These include enhancing the tricarboxylic acid (TCA) cycle, urea cycle, proline cycle, and glycerophospholipid metabolism, thereby promoting the oxidative utilization of fatty acids and proteins. Concurrently, the extract reduces inflammation and ameliorates oxidative stress and pain through the sphingolipid pathway and the NO/cGMP signaling pathway. We speculate that the bioactive constituents responsible for the anti-CFS effects include flavonoids (such as koburaside, gigantol, vanilloloside, and rutin), coumarins (such as scopoletin, samidin, and disenecionyl cis-khellactone), amide alkaloids, and bibenzyls. Representative compounds of the latter two classes include Tyramine, Dendrocandin B, Dendrocandin F, and Densiflorol A, which also exhibit potential therapeutic effects against CFS. Our research is anticipated to provide crucial data support and a theoretical foundation for elucidating the mechanism underlying the anti-CFS effects of the ethanolic extract of *D. officinale* and for the development of related therapeutic products.

## Data Availability

The original contributions presented in the study are included in the article/Supplementary Material, further inquiries can be directed to the corresponding authors.
