# Supplementary material for: Anti-fatigue mechanism of Dendrobium officinale pseudobulbs from different growth durations against chronic fatigue syndrome in mice and analysis of their active chemical components
Source: Front Cell Dev Biol. 2026 Apr 22;14:1797074. doi: 10.3389/fcell.2026.1797074 (PMC13143996; doi:10.3389/fcell.2026.1797074)
Supplement: Supplementary file 1 [file Table1.docx]

Supplementary Material

# Supplementary Tables

Table S1 丨 Plasma metabolites differentially abundant between the model group and normal group in mice with exercise-induced fatigue, and their responses to treatment with 1DOE and 3DOE*.*

| No. | Metabolites | m/z | Ion mode | Model vs. Normal | 3DOE vs. Model | *p* | Adjusted *q* (FDR) | VIP |
| --- | --- | --- | --- | --- | --- | --- | --- | --- |
| 1 | Hexanoyl-L-carnitine | 260.1853 | P | ↑ | ↓ | 0.0092 | 0.0208 | 1.918 |
| 2 | Octanoylcarnitine | 288.2168 | P | ↑ | ↓ | 0.0161 | 0.0283 | 1.811 |
| 3 | Acylcarnitine C20:2 | 452.3713 | P | ↑ | ↓ | 0.0195 | 0.0309 | 1.754 |
| 4 | O-Acylcarnitine | 258.1697 | P | ↑ | ↓ | 0.0278 | 0.0368 | 1.681 |
| 5 | L-Carnitine | 162.1123 | P | ↑ | ↓ | 0.0128 | 0.0249 | 1.562 |
| 6 | Acylcarnitine C20:5 | 446.3254 | P | ↑ | ↓ | 0.0414 | 0.0453 | 1.667 |
| 7 | Lysophosphatidylcholine LPC(16:0) | 496.3300 | P | ↑ | ↓ | 0.0087 | 0.0201 | 1.913 |
| 8 | LPC(17:1) | 508.3388 | P | ↑ | ↓ | 0.0301 | 0.0383 | 1.733 |
| 9 | LPC(22:5) | 570.3544 | P | ↓ | ↓ | 0.0126 | 0.0247 | 1.524 |
| 10 | PC(20:5/22:5) | 854.5677 | P | ↓ | ↑ | 0.0178 | 0.0295 | 1.353 |
| 11 | L-Dilinoleoyllecithin | 782.5681 | P | ↓ | ↓ | 0.0258 | 0.0356 | 1.238 |
| 12 | Sphingosine | 300.2893 | P | ↑ | ↓ | 0.0132 | 0.0254 | 1.725 |
| 13 | Sphinganine-phosphate | 382.2707 | P | ↑ | ↓ | 0.0442 | 0.0470 | 1.447 |
| 14 | Sphingosine 1-phosphate | 380.2555 | P | ↑ | ↓ | 0.0191 | 0.0306 | 1.404 |
| 15 | Oxoglutaric acid | 145.0140 | N | ↓ | ↓ | 4.835E-04 | 0.0047 | 1.614 |
| 16 | Citric acid | 191.0199 | N | ↓ | ↓ | 0.0013 | 0.0074 | 1.547 |
| 17 | D-(+)-Malic acid | 133.0140 | N | ↓ | ↓ | 0.0282 | 0.0370 | 1.208 |
| 18 | L-tyrosine | 180.0669 | N | ↓ | ↑ | 0.0160 | 0.0283 | 1.581 |
| 19 | Hydroxyphenyllactic acid | 181.0505 | N | ↓ | ↓ | 0.0039 | 0.0133 | 1.407 |
| 20 | Cytidine | 244.0921 | P | ↑ | ↓ | 0.0065 | 0.0173 | 1.787 |
| 21 | Thymine | 127.0496 | P | ↓ | ↓ | 0.0162 | 0.0283 | 1.370 |
| 22 | (R)-3-Hydroxybutyric acid | 103.0398 | N | ↓ | ↓ | 0.0219 | 0.0328 | 1.283 |
| 23 | Guanosine monophosphate | 364.0637 | P | ↓ | ↓ | 0.0249 | 0.0350 | 1.211 |
| 24 | Xanthosine | 283.0685 | N | ↓ | ↓ | 0.0426 | 0.0461 | 1.202 |
| 25 | Palmitoleic acid | 255.2312 | P | ↑ | ↓ | 0.0130 | 0.0253 | 1.868 |
| 26 | Arachidonic acid | 305.2471 | P | ↓ | ↑ | 0.0429 | 0.0462 | 1.441 |
| 27 | Linoleic acid | 279.2336 | N | ↓ | ↓ | 0.0427 | 0.0461 | 1.171 |
| 28 | Pyroglutamic acid | 147.0760 | P | ↑ | ↓ | 0.0206 | 0.0317 | 1.318 |
| 29 | L-5-Oxoproline | 130.0494 | P | ↑ | ↑ | 0.0186 | 0.0302 | 1.283 |
| 30 | Biotin | 245.0995 | P | ↓ | ↑ | 0.0018 | 0.0087 | 1.631 |
| 31 | 1-Pyrroline-5-carboxylic acid | 114.0543 | P | ↑ | ↑ | 0.0019 | 0.0091 | 1.585 |
| 32 | Taurine | 124.0071 | N | ↑ | ↓ | 0.0249 | 0.0350 | 1.511 |
| 33 | 2-Coumaric acid | 165.0542 | P | ↓ | ↑ | 0.0299 | 0.0381 | 1.364 |
| 34 | TG(50:7) | 843.6568 | P | ↓ | ↓ | 0.0265 | 0.0359 | 1.214 |
| 35 | Betaine | 118.0857 | P | ↑ | ↑ | 0.0480 | 0.0489 | 1.144 |
| 36 | Phe-His | 303.1413 | P | ↓ | ↑ | 5.624E-05 | 0.0019 | 2.008 |

Table S2 丨 Putatively annotated constituents differing between 1DOE and 3DOE

Annotation note. Table S2 summarizes putatively annotated candidate constituents highlighted by multivariate analysis to describe class-level and overall chemical-composition differences between 1DOE and 3DOE. Compound annotations were assigned based on accurate mass, MS/MS fragmentation behavior, retention behavior, and database/literature matching, and should therefore be interpreted as putative annotations rather than confirmed identifications based on authentic reference standards.

| Number | Rt/min | Compounds | Molecular Formula | Ion mode | m/z | Ontology |
| --- | --- | --- | --- | --- | --- | --- |
| 1 | 0.71 | (S)-malate | C_4_H_6_O_5_ | N | 133.014 | Organic acids |
| 2 | 0.71 | Arginine | C_6_H_14_N_4_O_2_ | P | 175.12 | Amino acids |
| 3 | 2.01 | Scopoletin | C_10_H_8_O_4_ | P | 193.05 | Coumarins |
| 4 | 2.04 | Deoxyadenosine | C_10_H_13_N_5_O_3_ | P | 252.11 | Nucleosides |
| 5 | 2.37 | Koaburaside | C_14_H_20_O_9_ | N | 331.103 | Phenolic compounds |
| 6 | 2.55 | Gigantol | C_16_H_18_O_4_ | P | 275.128 | Bibenzyls |
| 7 | 2.55 | Vanilloloside | C_14_H_20_O_8_ | N | 315.108 | Phenolic compounds |
| 8 | 3.2 | Salidroside | C_14_H_20_O_7_ | P | 301.129 | Phenolic compounds |
| 9 | 3.56 | Vicenin-2 | C_27_H_30_O_15_ | N | 593.151 | Flavonoids |
| 10 | 3.64 | Manglieside E | C_28_H_38_O_13_ | N | 581.223 | Lignans |
| 11 | 3.70 | Salicylamide | C_7_H_7_NO_2_ | N | 136.04 | Phenolic compounds |
| 12 | 3.75 | Schaftoside | C_26_H_28_O_14_ | N | 563.14 | Flavonoids |
| 13 | 3.84 | Vicenin-1 | C_26_H_28_O_14_ | N | 563.14 | Flavonoids |
| 14 | 3.86 | (+)-Lyoniresinol 9'-O-glucoside | C_28_H_38_O_13_ | N | 581.223 | Lignans |
| 15 | 3.89 | Coniferinoside | C_22_H_32_O_13_ | P | 505.192 | Phenylpropanoids |
| 16 | 3.91 | Apigenin-6-C-β-D-xylopyranosyl-8-C-α-L-arabinopyranoside (AXA) | C_25_H_26_O_13_ | N | 533.13 | Flavonoids |
| 17 | 3.93 | Rutin | C_27_H_30_O_16_ | N | 609.146 | Flavonoids |
| 18 | 4.01 | Isovitexin | C_21_H_20_O_10_ | N | 431.098 | Flavonoids |
| 19 | 4.02 | p-Cymene | C_10_H_14_ | P | 135.117 | Terpenoids |
| 20 | 4.05 | Quercetin-3-O-beta-D-galactoside | C_21_H_20_O_12_ | N | 463.088 | Flavonoids |
| 21 | 4.09 | Lyoniside | C_27_H_36_O_12_ | P | 553.229 | Lignans |
| 22 | 4.18 | Isoschaftoside | C_26_H_28_O_14_ | P | 565.156 | Flavonoids |
| 23 | 4.20 | Azelaic acid | C_9_H_16_O_4_ | N | 187.097 | Organic acids |
| 24 | 4.27 | Taxifolin | C_15_H_12_O_7_ | N | 303.05 | Flavonoids |
| 25 | 4.27 | Lirioresinol A | C_22_H_26_O_8_ | P | 419.171 | Lignans |
| 26 | 4.28 | Acanthoside B | C_28_H_36_O_13_ | N | 579.208 | Lignans |
| 27 | 4.43 | Pyridoxine | C_8_H_11_NO_3_ | P | 170.082 | Others |
| 28 | 4.512 | Dihydrokaempferol | C_15_H_12_O_6_ | N | 287.056 | Flavonoids |
| 29 | 4.63 | N-Dihydroferuloyltyramine | C_18_H_21_NO_4_ | P | 316.155 | Alkaloids |
| 30 | 4.73 | Coumaroyl tyramine | C_17_H_17_NO_3_ | P | 284.129 | Alkaloids |
| 31 | 4.83 | Feruloyltyramine | C_18_H_19_NO_4_ | P | 314.139 | Alkaloids |
| 32 | 4.89 | Eriodictyol | C_15_H_12_O_6_ | N | 287.056 | Flavonoids |
| 33 | 4.94 | Quercetin | C_15_H_10_O_7_ | N | 301.035 | Flavonoids |
| 34 | 5.01 | Densiflorol A | C_16_H_16_O_4_ | P | 273.113 | Bibenzyls |
| 35 | 5.08 | Aquilegiolide | C_8_H_8_O_3_ | N | 151.04 | Others |
| 36 | 5.27 | Naringenin | C_15_H_12_O_5_ | N | 271.061 | Flavonoids |
| 37 | 5.27 | Apigenin | C_15_H_10_O_5_ | N | 269.045 | Flavonoids |
| 38 | 5.51 | Homoeriodictyol | C_16_H_14_O_6_ | N | 301.071 | Flavonoids |
| 39 | 6.06 | Dendrocandin F | C_32_H_32_O_8_ | P | 545.218 | Bibenzyls |
| 40 | 6.07 | Tyramine | C_8_H_11_NO | P | 138.092 | Alkaloids |
| 41 | 6.47 | Dendrocandin B | C_27_H_30_O_8_ | P | 461.218 | Bibenzyls |
| 42 | 6.55 | LPC(18:2) | C_26_H_50_NO_7_P | P | 520.34 | Lipids |
| 43 | 6.63 | 3'-Senecioyl khellactone | C_19_H_20_O_6_ | P | 345.134 | Coumarins |
| 44 | 6.63 | Gingerglycolipid B | C_33_H_58_O_14_ | P | 759.526 | Lipids |
| 45 | 6.91 | LPC(14:0) | C_22_H_46_NO_7_P | P | 468.309 | Lipids |
| 46 | 6.98 | LPC(18:0) | C_26_H_54_NO_7_P | P | 524.372 | Lipids |
| 47 | 7.96 | Disenecionyl cis-khellactone | C_24_H_26_O_7_ | P | 427.176 | Coumarins |
| 48 | 7.97 | Samidin | C_21_H_22_O_7_ | P | 387.144 | Coumarins |
| 49 | 8.06 | Arachidic acid | C_20_H_40_O_2_ | N | 311.295 | Fatty acids |
| 50 | 8.18 | LPC(18:3) | C_26_H_48_NO_7_P | P | 518.325 | Lipids |
| 51 | 8.33 | Pinolenic acid | C_18_H_30_O_2_ | N | 277.217 | Fatty acids |
| 52 | 8.76 | Linoleic acid | C_18_H_32_O_2_ | N | 279.232 | Fatty acids |
| 53 | 9.34 | 7-Oxostigmasterol | C_29_H_46_O_2_ | P | 427.358 | Terpenoids |
| 54 | 9.56 | Schleicherastatin 5 | C_29_H_48_O_3_ | P | 445.368 | Terpenoids |
